# Adalimumab induced mononeuritis multiplex in a patient with refractory rheumatoid arthritis: a case report

**DOI:** 10.1186/1757-1626-1-287

**Published:** 2008-10-30

**Authors:** Ashima Makol, Madhusudan Grover

**Affiliations:** 1 B-301 Clinical Center, Michigan State University, East Lansing, MI-48824, USA

## Abstract

**Background:**

Anti tumor necrosis factor agents are a valuable addition to the armamentarium against rheumatoid arthritis but have some serious side effects which clinicians should be aware about.

**Case presentation:**

We present a case of a 54 year old Caucasian male with refractory rheumatoid arthritis who developed mononeuritis multiplex four weeks after starting adalimumab therapy.

**Conclusion:**

Complete resolution of neurological and electromyography findings was seen upon stopping therapy. These agents can be a double-edged sword in the management of rheumatological illnesses. Though they help treat refractory disease, their potential side effects can include mononeuritis multiplex which can be recognized by means of clinical features, electromyography and nerve biopsy as depicted in our case.

## Background

Anti tumor necrosis factor (TNF) agents are emerging in the frontline management of rheumatoid arthritis (RA) in the current era of biologicals. They have considerable side effects including infections, malignancies, and hypersensitivity reactions. Vasculitis leading to mononeuritis multiplex (MM) is a rare but potentially serious complication of RA and has been previously reported after infliximab therapy [1]. We present a patient with RA who developed MM on adalimumab therapy.

## Case presentation

A 54 year old Caucasian male with a history of RA for last 25 years refractory to multiple medications including gold, methotrexate, cyclosporine and leflunomide was started on adalimumab 4 weeks prior to presentation. He presented with progressively increasing pain, parasthesias and numbness of bilateral upper and lower extremities of 2 weeks duration followed by a right wrist and left foot drop. He lost 25 lbs weight during this period. He denied any history of smoking or alcohol intake. He was on permanent disability secondary to severe destructive joint disease with his RA. He did not have any prior neurological problems. Other medical problems included hypertension and gastroesophageal reflux disease for which he was on lisinopril and famotidine respectively. He had tonsillectomy and adenoidectomy as a child. There was no significant family history. He weighed 100 lbs and was 5 foot 7 inch tall. Physical examination revealed ulnar deviation with swan neck and boutonniere deformities of the fingers bilaterally. Paresis of dorsiflexion and eversion of the right foot and dorsiflexion of the left wrist were also present. Rheumatoid factor titer was high at 337 IU/ml, ESR and CRP elevated at 84 mm/h and 5.9 mg/dl respectively and C4 level was low at 12 indicating high disease activity. A work up for peripheral neuropathy revealed normal TSH, Vitamin B, CPK and Acetyl-L-carnitine levels. Monospot test, VDRL, HIV antibody, P- and C-ANCA, ANA, anti double-stranded DNA, heavy metal screen and a paraneoplastic panel were all negative. Serologies for hepatitis and borrelia were negative. MRI of the spine ruled out cord lesion. Electromyography (EMG) of the left upper extremity and right lower extremity showed axonal neuropathy with spontaneous activity and sharp wave fibrillation potentials. Sural nerve biopsy revealed focal segmental necrotizing arteritis with fibrinoid necrosis, loss of thick fibres and axonal degeneration consistent with MM. Adalimumab was stopped and high dose methylprednisolone (40 mg q12h) and cyclosporine (25 mg q12h) were started which led to resolution of symptoms. He was discharged on oral prednisone and cyclophosphamide. Resolution of neurological symptoms and EMG abnormalities were noted at three months follow up. He regained 18 lbs of his lost weight during these 3 months.

## Discussion

MM is a painful, asymmetric, asynchronous sensory and motor peripheral neuropathy involving at least 2 separate nerves and often associated with constitutional features including fatigue and significant weight loss. It is a rare but known complication of long-standing RA secondary to vasculitic neuropathy. Anti TNF agents have been reported in successful treatment of neuropathy in RA. However, flare up of neuropathy has also been reported with these agents[2,3]. In particular, infliximab therapy has been associated with multifocal motor neuropathy with conduction block as well as axonal sensory polyneuropathy which reversed upon discontinuation of infliximab and intravenous gammaglobulin treatment [2]. Mechanisms proposed include induction of auto-antibodies and T cell mediated cytokines. MS patients treated with these agents showed an increase in gadolinium-enhancing lesions on MRI[4] and the number of flares[5] indicating that TNF-α might have a protective role against inflammation and demyelination. Though a progression of RA or a de novo onset of vasculitis cannot be excluded, the temporal association of symptoms on starting adalimumab and their subsequent disappearance on stopping, and a negative work up for other possible causes of MM, is indicative of a drug induced phenomenon.

## Conclusion

TNF antagonists represent a major breakthrough in the treatment of rheumatologic diseases but are not without potentially serious adverse effects. To our knowledge this is the first report of association of MM with adalimumab. Future studies are needed to assess the possible association of anti-TNF-agents with MM in order to assist clinicians with appropriate clinical decision-making.

## Abbreviations

ESR: Erythrocyte sedimentation rate; CRP: C- reactive protein; C4: Complement 4; TSH: Thyroid stimulating hormone; CPK: Creatine Phosphokinase; VDRL: Venereal Disease Research Laboratory; HIV: Human Immunodeficiency Virus; ANCA: Anti-neutrophil cytoplasmic antibodies; ANA: Anti-nuclear antibodies; DNA: Deoxyribonucleic Acid; MRI: Magnetic Resonance Imaging; MS: Multiple Sclerosis.

## Consent

Written informed consent was obtained from the patient for publication of this case report and a copy of the written consent is available for review by the Editor-in-Chief of this journal.

## Competing interests

The authors declare that they have no competing interests.

## Authors' contributions

AM collected clinical data, reviewed literature on the topic and drafted the manuscript and MG critically revised the manuscript. All authors read and approved the final manuscript.
